# Intranasal Vaccination with *Chlamydia pneumoniae* Induces Cross-Species Immunity against Genital *Chlamydia muridarum* Challenge in Mice

**DOI:** 10.1371/journal.pone.0064917

**Published:** 2013-05-31

**Authors:** Srikanth Manam, Bharat K. R. Chaganty, Shankar Jaikishan Evani, Mark T. Zafiratos, Anand K. Ramasubramanian, Bernard P. Arulanandam, Ashlesh K. Murthy

**Affiliations:** 1 Department of Pathology, Midwestern University, Downers Grove, Illinois, United States of America; 2 South Texas Center for Emerging Infectious Diseases, Department of Biology, The University of Texas at San Antonio, San Antonio, Texas, United States of America; Duke University Medical Center, United States of America

## Abstract

*Chlamydia trachomatis* is the most common bacterial sexually transmitted disease in the world and specifically in the United States, with the highest incidence in age-groups 14–19 years. In a subset of females, the *C. trachomatis* genital infection leads to serious pathological sequelae including pelvic inflammatory disease, ectopic pregnancy, and infertility. *Chlamydia pneumoniae*, another member of the same genus, is a common cause of community acquired respiratory infection with significant number of children aged 5–14 yr displaying sero-conversion. Since these bacteriae share several antigenic determinants, we evaluated whether intranasal immunization with live *C. pneumoniae* (1×10^6^ inclusion forming units; IFU) in 5 week old female C57BL/6 mice would induce cross-species protection against subsequent intravaginal challenge with *Chlamydia muridarum* (5×10^4^ IFU), which causes a similar genital infection and pathology in mice as *C. trachomatis* in humans. Mice vaccinated intranasally with live *C. pneumoniae*, but not mock (PBS) immunized animals, displayed high levels of splenic cellular antigen-specific IFN-γ production and serum antibody response against *C. muridarum and C. trachomatis*. Mice vaccinated with *C. pneumoniae* displayed a significant reduction in the vaginal *C. muridarum* shedding as early as day 12 after secondary i.vag. challenge compared to PBS (mock) immunized mice. At day 19 after *C. muridarum* challenge, 100% of *C. pneumoniae* vaccinated mice had cleared the infection compared to none (0%) of the mock immunized mice, which cleared the infection by day 27. At day 80 after *C. muridarum* challenge, *C. pneumoniae* vaccinated mice displayed a significant reduction in the incidence (50%) and degree of hydrosalpinx compared to mock immunized animals (100%). These results suggest that respiratory *C. pneumoniae* infection induces accelerated chlamydial clearance and reduction of oviduct pathology following genital *C. muridarum* challenge, and may have important implications to the *C. trachomatis*-induced reproductive disease in humans.

## Introduction

*Chlamydia trachomatis* is the leading cause of bacterial sexually transmitted infection (STI), with approximately 90 million new cases detected annually worldwide [1]. The greatest incidence of infection is in the 14–19 year age group [2]. The infection is easily treatable with available antimicrobials [1], [3]. However, repeated infections with the same or a different serovar occur commonly [4]. According to the Centers for Disease Control (2004), approximately 20–40% of women with past history of *C. trachomatis* infection(s) in the lower genital tract have been reported to develop serious sequelae such as pelvic inflammatory disease (PID). Subsets of patients with PID develop complications such as ectopic pregnancy and tubal infertility.

It is unclear why the reproductive sequelae develop only in a subset of women who contract chlamydial genital infections. Substantial research has been conducted to understand the immunity and pathogenesis relating to chlamydial STI using intravaginal *C. muridarum* infection in a mouse model that reasonably mimics the genital *C. trachomatis* infection and pathogenesis in humans [1], [3]. While natural immunity confers resistance to reinfection, at least a subset of immune responses has been shown to be instrumental in the causation of pathologies. For example, several factors including activation of toll-like receptor-2 [5], neutrophil and matrix metalloprotease responses [6], [7], and CD8^+^ T cells and TNF-α production [8] have been shown to contribute to the development of chlamydial pathological sequelae in the mouse model. Thus, the development of varying degrees of immune responses among individuals in a population, and pathological responses specifically, may determine the development of disease sequelae in some but not all infected women. Furthermore, chlamydial STI may resolve spontaneously after several months due to natural immunity, or be detected and treated early with antimicrobials [1]. In the former scenario, a robust immune response may act as a double-edged sword, assisting in the spontaneous clearance of infection while also promoting development of pathologies [9]. This line of thought has led to an emphasis on the induction of a protective, while eliminating pathogenic, immune response for anti-chlamydial vaccine development [10]. The latter scenario supports the “arrested immunity” hypothesis [11], which states that early detection and treatment of the infection prevents the development of a robust immune response. The consequences may be two-fold; increased susceptibility to infection upon subsequent exposure, and reduced propensity to develop pathological sequelae. In fact, the introduction of robust screening programs for chlamydial STI has been associated with dramatically increased prevalence rates of genital chlamydial infections and a parallel reduction in the prevalence of *Chlamydia*-associated PID [11]. Irrespectively, both these scenarios are supported by the principle that host immune response against chlamydial infection is capable of inducing protective immunity, but also contributes to pathogenesis.

While the aforementioned scenarios are fully plausible and likely contribute, we suggest that an additional distinct variable typically operational in human populations may be capable of affecting the course of *C. trachomatis* infection and pathological sequelae in human populations. *Chlamydia pneumoniae* is the causative agent of respiratory disease in humans and one of the major causes of community acquired pneumonia [12]. Beginning with a low prevalence in children under 5 years age, the prevalence increases dramatically to over 40% between 5–14 years age [12]. Approximately 50% of persons at age 20, and 75% of elderly have detectable antibody against *C. pneumoniae*. Importantly, a significant proportion of children display antibodies against *C. pneumoniae* before the onset of sexual activity and exposure to *C. trachomatis* STI. Comparative analysis of the genomes has revealed a very high degree of synteny between *C. pneumoniae* and *C. trachomatis* genomes [13], suggesting the likelihood of several shared antigenic determinants. Therefore, the immune responses induced by one pathogen may mediate cross-species protective immunity or pathogenic responses against the other. Therefore, we hypothesized that intranasal immunization with live *C. pneumoniae* will affect the course of infection and pathological sequelae following genital *C. trachomatis* challenge.

We tested this hypothesis in the mouse model by comparing the effect of intranasal live *C. pneumoniae* AR39 immunization on the course of vaginal infection and oviduct pathological sequelae following intravaginal *C. muridarum* challenge in C57BL/6 mice.

## Materials and Methods

### Ethics Statement

All animal experiments were performed in compliance with the Animal Welfare Act, the U.S. Public Health Service Policy on Humane Care and Use of Laboratory Animals and “Guide for the Care and Use of Laboratory Animals” published by the National Research Council. Animal work was done in accordance with the guidelines set forth by the Institutional Animal Care and Use Committee (IACUC) at Midwestern University (Animal welfare assurance number A3048-01 and IACUC protocol number 2088) and at The University of Texas at San Antonio (Animal welfare assurance number A3592-01 and IACUC protocol number MU012). The respective IACUC at Midwestern University and the University of Texas at San Antonio specifically approved this study.

### Bacteria

*Chlamydia muridarum* Nigg and *Chlamydia trachomatis* serovar D were grown separately on confluent HeLa cell monolayers as described previously [14], [15]. *Chlamydia pneumoniae* was grown on confluent HEp-2 cell monolayers as described previously [16]. The infected cells were lysed by sonication and elementary bodies (EBs) purified on Renografin gradients. Aliquots of bacteria were stored at −70°C in sucrose–phosphate–glutamine (SPG) buffer.

### Mice

Four-to-six week old female C57BL/6 mice were used for all experiments. The mice were purchased from National Cancer Institute (Bethesda, MD) and housed at Midwestern University (MWU) and The University of Texas at San Antonio (UTSA). Animal care and experimental procedures were performed at MWU and UTSA in compliance with the respective Institutional Animal Care and Use Committee guidelines.

### Intranasal Immunization

Groups of mice were anesthetized on day 0 with inhalational 3% isofluorane and injected intranasally with 25 µl per mouse of 1× PBS containing 1×10^6^ IFU of *C. pneumoniae* AR39 (*C. pne*). One group of mice receiving PBS alone (mock) served as negative control for the experiments. In experiments to evaluate protective immunity against pathology, one group of mice receiving 0.5×10^3^ IFU of *C. muridarum* (*C. mur*) on day 0 only served as positive control. The usage of the respective inocula for *C. pne* and *C. mur* is consistent with well-established mouse models of intranasal infection with these pathogens [16], [17]. Additionally, one group of mice receiving i.n. live *C. pne* immunization followed by mock (PBS) genital challenge was used to evaluate any contribution of the immunization per se towards disease development in the genital tract.

### Splenic Cellular Antigen-specific Cytokine Responses

Spleens were removed 14 days after initial intranasal immunization and single cell suspensions prepared. Collected splenocytes [10^6^/well] were incubated for 72 h with 10^5^ IFU of UV-inactivated *C. mur* or *C. tra*, or 1 µg of an unrelated antigen bovine serum albumin (BSA), or in media alone in 96-well culture plates. Supernatants were assayed for levels of IFN-γ using ELISA kits (eBioscience, San Diego, CA) according to manufacturer’s instructions. Absorbance at 630 nm was measured using a Multiskan FC ELISA microplate reader (Thermoscientific Corp, Rockford, IL).

### Detection of Antibody Levels by ELISA

On day 50 following intranasal immunization, animals were bled, sera prepared and analyzed by ELISA as described previously [14]. Microtiter plates (96-well) were coated overnight with 10^5^ IFU per well of *C. mur* or *C. tra*, or 1 µg of an unrelated antigen BSA, or PBS alone in sodium bicarbonate buffer (pH 9.5). Serial dilutions of serum were added to wells followed by goat anti-mouse total Ig (Southern Biotech, Birmingham, AL). After washing, horse radish peroxidase substrate (Sigma, St. Louis, MO) was added for color development and absorbance (O.D.) at 630 nm monitored using a Multiskan FC ELISA microplate reader (Thermoscientific Corp, Rockford, IL). Reciprocal serum dilutions corresponding to 50% maximal binding were used to obtain titers.

### Vaginal Infection, Determination of Chlamydial Shedding and UGT Pathology

We confirmed that no chlamydial organisms could be recovered from the lungs of either *C. pne* or *C. mur* infected mice at day 45 after the intranasal immunization (data not shown). Mice were challenged i.vag. with 5×10^4^ IFU of *C. muridarum* 60 days following intranasal immunization. This was considered day 0 for the challenge infection. Ten and three days prior to intravaginal *C. mur* challenge, mice were treated with 2.5 mg of Depo-Provera (Upjohn, Kalamazoo, MI). Vaginal swab material was collected at the indicated days after challenge and chlamydial enumeration conducted by plating swab material on HeLa cell monolayers followed by immunofluorescent staining [14]. On day 80 after challenge, mice were euthanized, the genital tracts removed, placed next to a standard metric ruler, photographed and the greatest cross-sectional diameter measured for each oviduct, and reported individually and as mean ± SEM in a group, as also described previously [8]. Based on previous studies, we have found, that oviducts of age-matched, depo-progesterone treated but uninfected female mice measure ≤0.5 mm. Therefore, a threshold of 0.5 mm was used to distinguish normal from dilated oviducts. The enumeration of chlamydial counts and oviduct measurements was conducted in a blinded fashion.

### Statistics

Sigma Stat (Systat Software Inc., San Jose, CA) was used to perform all tests of significance. ANOVA was used to compare between multiple groups for cytokine and antibody response, vaginal chlamydial shedding and dilatation of hydrosalpinx. For vaginal chlamydial shedding, ANOVA with all pairwise multiple comparison procedures (Holm-Sidak method) was used to compare multiple groups at each time-point. The number of mice shedding *Chlamydia* at each time point in a group, versus those that did not, were added over the monitored period and the sums were compared between groups using Fisher’s exact test. Fisher’s exact test also was used to compare the incidence of hydrosalpinx. P<0.05 was considered statistically significant. All experiments were repeated at least twice, and each experiment was analyzed independently. Where oviduct diameter data is shown as a composite of two experiments, the indicated significant difference holds true when the experiments are analyzed individually.

## Results

### Antigen-specific Immune Responses after Intranasal Live *C. pneumoniae* Immunization

**Figure 1:**
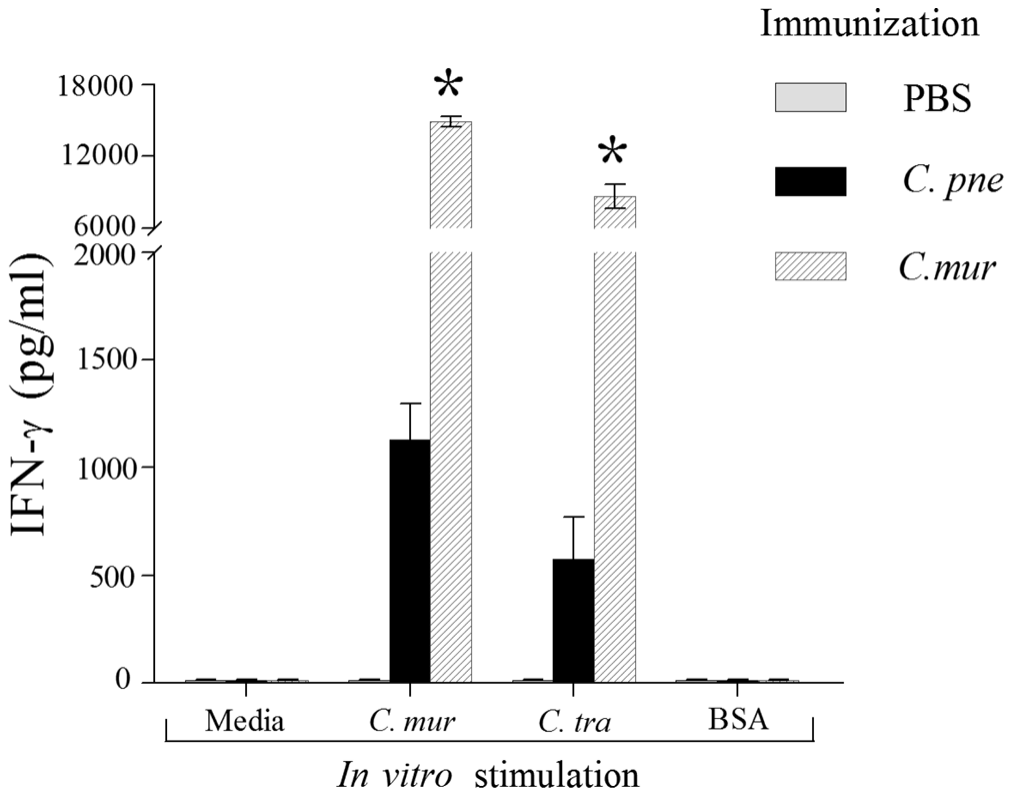
Splenic cellular antigen-specific IFN-γ response following immunization. C57BL/6 mice (3 mice/group) were treated i.n. with *C. pneumoniae* (1×10^6^ IFU/mouse), or *C. muridarum* (0.5×10^3^ IFU/mouse) in 25 µl of PBS, or PBS alone (mock). On day 14, animals were euthanized and splenocytes were tested by ELISA for antigen-specific IFN-γ production against *C. mur*, *C. tra,* or the unrelated antigen BSA. Cells cultured in media alone were used to evaluate baseline cytokine production from isolated splenocytes. Results are expressed as mean ± SEM of IFN-γ production per culture condition. *Significant (P<0.05, ANOVA) differences in IFN-γ production from splenocytes of *C. pne* versus *C. mur* immunized mice upon stimulation with *C. mur* or *C. tra*. Results are representative of two independent experiments.

Immunity against genital chlamydial infection has been shown to involve antigen-specific IFN-γ producing cellular responses and antibody production [1], [3], [18]. Therefore, we evaluated these immune responses after i.n. immunization with *C. pne* or *C. mur*. On day 14 after initial intranasal *C. pne, C. mur,* or PBS (mock) immunization, mice were euthanized; spleens were removed and single cell suspensions made. Splenocytes (10^6^ cells/well) were stimulated for 72 h with *C. mur* or *C. tra* or with an unrelated antigen BSA, or incubated in media alone. As shown in Fig. 1, *C. pne* immunized mice displayed high levels of IFN-γ response against *C. mur* and *C. tra*. Mice immunized i.n. with *C. mur* displayed significantly greater IFN-γ production when stimulated *in vitro* with either *C. mur* or *C. tra* when compared to *C. pne* immunized mice. Minimal IFN-γ response was produced against chlamydial antigens by cells from mice immunized with PBS, and from cells obtained from *C. pne, C. mur* or PBS immunized groups incubated with the unrelated antigen BSA, or media alone. Splenocytes from *C. pne* immunized mice expectedly displayed significantly greater IFN-γ production upon stimulation with *C. pne* as compared to any other treatment (data not shown).

**Figure 2:**
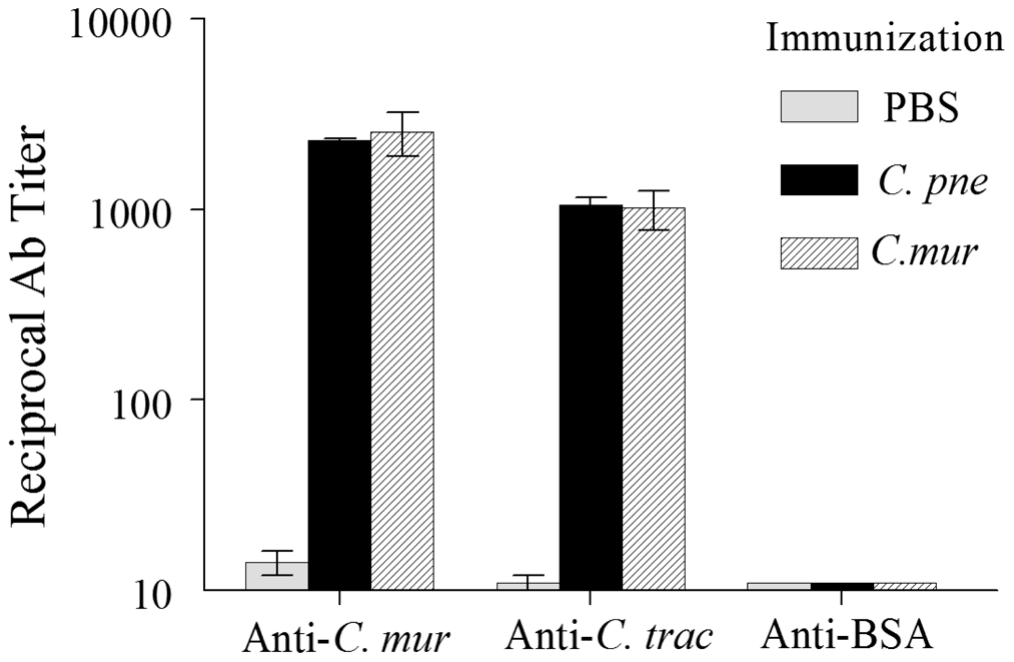
Serum antibody response following immunization. C57BL/6 mice (3 mice/group) were treated i.n. with *C. pneumoniae* (1×10^6^ IFU/mouse), or *C. muridarum* (0.5×10^3^ IFU/mouse) in 25 µl of PBS, or PBS alone (mock). On day 50, animals were bled and serum total antibody levels against *C. mur*, *C. tra*, or the unrelated antigen BSA were analyzed by ELISA. Results are expressed as mean ± SEM of reciprocal serum dilutions corresponding to 50% maximal binding. Results are representative of two independent experiments.

The serum total antibody responses also were measured against *C. mur* and *C. tra* at day 50 following the intranasal immunization. As shown in Fig. 2, high and comparable levels of cross-species anti-*C. mur* and anti-*C. tra* total Ab response was induced by both *C. pne* and *C. mur* immunization. There was minimal binding in plates coated with an unrelated antigen BSA, indicating the specificity of these responses against chlamydial antigens. Serum anti-*C. pne* total antibody levels expectedly were significantly greater than cross-reactive antibodies against *C. mur* or *C. tra* (data not shown). Collectively, these results demonstrate that i.n. immunization with live *C. pne* induce antigen specific IFN-γ and antibody response against *C. muridarum* and *C. trachomatis*.

### Vaginal Bacterial Clearance following Chlamydial Challenge in Immunized Mice

**Figure 3:**
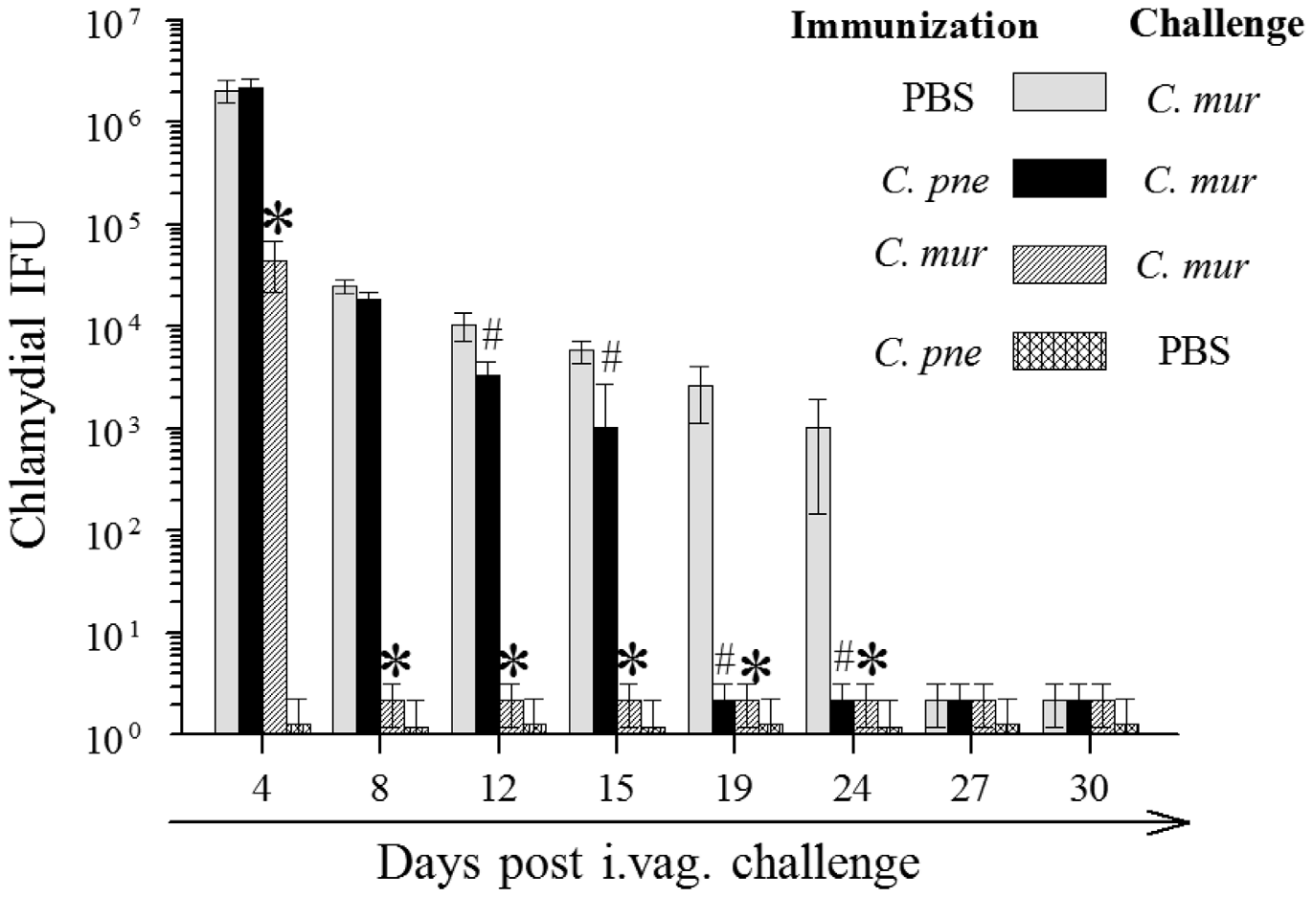
Bacterial clearance after intravaginal *C.* *muridarum* challenge in immunized animals. C57BL/6 mice (3 mice/group) were treated i.n. with *C. pneumoniae* (1×10^6^ IFU/mouse), or *C. muridarum* (0.5×10^3^ IFU/mouse) in 25 µl of PBS, or PBS alone (mock). The mice were challenged 60 days later intravaginally with 5×10^4^ IFU of *C. mur* in 10 µl of PBS. One group of *C. pne* immunized mice were mock challenged intravaginally with 10 µl of PBS alone. At the indicated days following challenge, numbers of chlamydial inclusion forming units (IFU) recovered from vaginal swabs was measured. Results are expressed as mean ± SEM of chlamydial shedding per group at each time point. Significant (P = 0.013, ANOVA all pairwise multiple pairwise procedures using Holm-Sidak method) difference between *C. pne*, *C. mur*, and PBS (mock) immunized groups. Within this test, significant differences also were detected between * mouse groups immunized with *C. mur* compared to to PBS (mock) and between # mouse groups immunized with *C. pne* compared to PBS (mock). Results are representative of two independent experiments.

The immunized animals were rested for one month following the i.n. immunization and challenged i.vag. with 5×10^4^ IFU of *C. muridarum*. The vaginal chlamydial shedding was monitored on the indicated days for a period of 30 days following bacterial challenge. As shown in Fig. 3 and Table 1, PBS (mock) immunized mice displayed high levels of chlamydial shedding on day 4 after inoculation, and displayed progressive reduction in bacterial shedding, with complete resolution by day 27 after inoculation. *C. pne* immunized mice displayed comparable bacterial shedding on days 4 and 8, followed by significantly reduced shedding on day 12 and subsequent time periods when compared to mock immunized animals. All (100%) *C. pne* immunized mice displayed complete resolution of infection by day 19 after inoculation, and chlamydial shedding in this group was significantly reduced over the course of infection when compared to mock immunized animals. As expected, *C. mur* immunized mice exhibited significantly reduced chlamydial shedding as early as day 4, and all mice had completely resolved the infection by day 8 after inoculation. *C. mur* immunized mice displayed significant reduction in shedding when compared to mock (PBS) immunized animals. As also expected, *C. pne* immunized and mock (PBS) challenged mice did not display shedding of either *C. pne* or *C. mur* from the vagina. These results suggest that i.n. immunization with live *C. pne* induces reduced chlamydial shedding and early bacterial clearance following i. vag. *C. muridarum* challenge.

**Table 1:** Bacterial clearance after intravaginal *C. muridarum* challenge in immunized animals.

i.n infection/i.vag. challenge	% of mice shedding *Chlamydia* from the vagina Days after i.vag. challenge							
	4	8	12	15	19	24	27	30
PBS/*C. mur*	100	100	100	100	100	33	0	0
*# C. pne*/*C. mur*	100	100	67	67	0	0	0	0
*C. mur/C. mur*	33	0	0	0	0	0	0	0
*C. pne*/PBS	0	0	0	0	0	0	0	0

The percentage of mice within a group (*n* = 6) shedding *Chlamydia* at each time point was added over the entire monitoring period, and the sum was compared between groups.

* Significant (P<0.05, Fisher’s exact test) differences between mouse groups immunized with PBS (mock) compared to *C. pne* and PBS (mock) compared to *C. mur*.

# Significant (P<0.05, Fisher’s exact test) differences between mouse groups immunized with *C. pne* compared to *C. mur*. Results are representative of two independent experiments.

### Upper Genital Tract Pathology following Challenge in Immunized Mice

The immunized/challenged mice were rested until day 80 after challenge and upper genital tract pathology was evaluated. Our previous extensive studies [8], [14], [18]–[21] have demonstrated the suitability of this time-period for analyses of upper genital tract sequelae induced by genital *C. muridarum* infection in mice. The macroscopic pathology was evaluated based on the presence of hydrosalpinx, and is reported as the percentage of mice displaying hydrosalpinx bilateral, unilateral, and total hydrosalpinx at day 80 after chlamydial challenge (Table 2). Hydrosalpinx was observed in 100% (33% bilateral, 67% unilateral) of mock (PBS) immunized mice. In comparison, *C. pne* immunized mice displayed significant reduction in the incidence of hydrosalpinx by 50% (17% bilateral; 33% unilateral). As shown also in several previous studies [3], pathology was minimal in *C. mur* immunized group and only 16% (8% bilateral; 8% unilateral) displayed hydrosalpinx. The incidence of hydrosalpinx in *C. mur* immunized group was significantly reduced from that in mock (PBS) immunized mice, but not from that in *C. pne* immunized group. *Chlamydia pneumoniae* immunized mice that were not challenged intravaginally with *C. mur* displayed normal oviducts, suggesting that the intranasal *C. pne* immunization per se did not affect the oviduct.

**Table 2:** The incidence of oviduct pathology after intravaginal *C. muridarum* challenge in immunized animals.

i.n infection/i.vag. challenge	% of mice displaying hydrosalpinx Day 80 after i.vag. challenge		
	B	U	T
PBS/*C. mur*	33	67	100
*C. pne*/*C. mur*	17	33	50
*C. mur/C. mur*	8	8	16
*C. pne*/PBS	0	0	0

The number of mice within a group (*n* = 6) developing hydrosalpinx was compared between groups.

* Significant (P<0.05, Fisher’s exact test) differences between mouse groups immunized with PBS (mock) compared to *C. pne* and PBS (mock) compared to *C. mur*. Results are a composite of two independent experiments and thus reflect 12 mice per group.

**Figure 4:**
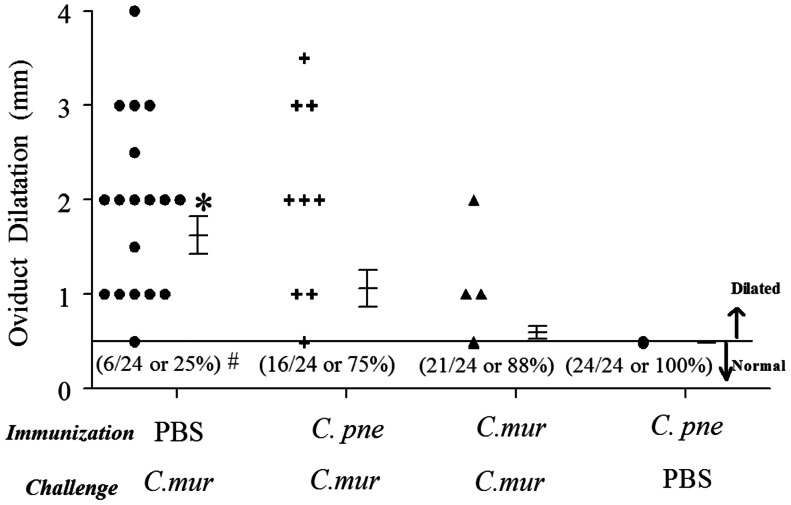
The incidence and severity of oviduct pathology after intravaginal *C.* *muridarum* challenge in immunized animals. C57BL/6 mice (3 mice/group) were treated i.n. with *C. pneumoniae* (1×10^6^ IFU/mouse), or *C. muridarum* (0.5×10^3^ IFU/mouse) in 25 µl of PBS, or PBS alone (mock). The mice were challenged 60 days later intravaginally with 5×10^4^ IFU of *C. mur* in 10 µl of PBS. One group of *C. pne* immunized mice was mock challenged intravaginally with 10 µl of PBS alone. Mice were euthanized, genital tract tissues collected, and macroscopic oviduct dilatation was measured on day 80 after challenge. Each individual marker represents one oviduct and the mean ± SEM of greatest cross-sectional oviduct diameter per group of mice also is shown. The number of normal oviducts (numerator) and the total number of oviducts evaluated (denominator), and the percentage of normal oviducts per respective group of mice have been indicated in parentheses. * Significant (P<0.05, ANOVA) difference in the degree of oviduct dilatation in *C. mur* challenged mice immunized previously with PBS (mock) compared to *C. pne*, and between mice immunized previously with PBS (mock) compared to *C. mur*. # Significant (P<0.05, Fisher’s exact test) difference in the incidence of oviduct dilatation in *C. mur* challenged mice immunized previously with PBS (mock) compared to *C. pne*, and in mice immunized previously with PBS (mock) compared to *C. mur*. Results are a composite of two independent experiments and thus reflect 24 oviducts per group.

We also quantified the oviduct dilatation by taking measurements of the greatest cross-sectional diameter of each oviduct, since the degree of oviduct dilatation may be an indicator of the severity of pathology. As shown in Fig. 4, 75% of oviducts in PBS immunized mice were dilated, and displayed a high level of dilatation. In comparison, significantly fewer (25%) oviducts were dilated in *C. pne* immunized mice, with a significant reduction in the degree of oviduct dilation when compared to mock (PBS) immunized animals. Additionally, *C. muridarum* immunized mice displayed further reduction, albeit not statistically different, in the incidence (12% oviducts) and degree of oviduct dilation when compared to *C. pne* immunized animals. All *Chlamydia pneumoniae* immunized mice that were not challenged intravaginally with *C. mur* displayed normal oviduct diameters. We analyzed these tissues further microscopically, but the cellular infiltration at 80 days after genital challenge was minimal (data not shown). Collectively, these results suggest that intranasal live *C. pne* immunization induces robust protective immunity, close to that induced by i.n. live *C. mur* immunization, against oviduct pathological sequelae following genital *C. mur* challenge.

## Discussion

We provide evidence that intranasal infection with *Chlamydia pneumoniae* induces cross-species antigen-specific IFN-γ and antibody response against *Chlamydia muridarum* and *Chlamydia trachomatis*. Furthermore, the i.n. *C. pneumoniae* infection induces significantly accelerated chlamydial clearance, and significant reduction in the incidence and degree of oviduct pathological sequelae following i.vag. *C. muridarum* challenge.

Cross-reactive immune responses in *C. pneumoniae* immunized mice induced significant reduction in chlamydial shedding and earlier resolution of the i.vag. *C. mur* infection compared to mock (PBS) immunized animals. To this end, we found a high level of *C. mur*-specific IFN-γ production from splenocytes of mice immunized previously with *C. pneumoniae*. Although there was accelerated resolution of the intravaginal challenge infection, all (100%) of *C. pneumoniae* immunized mice got infected upon i.vag. *C. mur* challenge and shed high levels of *C. mur* at days 4 and 8 after challenge, comparable to mock (PBS) immunized mice. This was in contrast to mice immunized i.n. with *C. mur* which displayed significant early resistance to the challenge, with 67% displaying no chlamydial shedding as early as day 4, and all (100%) of the mice resolving the infection by day 8 after i.vag. challenge. The differences in the level of protective immunity observed between i.n. *C. pne* versus *C. mur* immunized groups correlated with the differences in levels of splenic antigen-specific IFN-γ production. The production of *Chlamydia*-specific IFN-γ has been shown to be crucial in mediating enhanced clearance and protective immunity against *C. mur* challenge [1], [3], [18].

The total antibody levels against either *C. mur* were comparable between mice immunized i.n. with *C. pne* or *C. mur*, and did not correlate with the differences in clearance or pathology following i. vag. *C. mur* challenge. However, previous reports from the mouse model of *C. mur* genital infection have suggested an important role for antibody in inducing early resistance to reinfection [22]. Among several chlamydial antigens evaluated till date as putative vaccine candidates, only antibody against the chlamydial major outer membrane protein (MOMP) has been shown to be efficacious in neutralizing chlamydial infectivity [23]–[25]. Additionally, it has been shown that MOMP-induced neutralizing efficacy is dependent on the three dimensional conformation of this protein [26]. MOMP is the basis of serovar differentiation within *C. trachomatis*, and expectedly MOMP displays significant variation in amino acid sequence between different species of *Chlamydia* [27]. Therefore, even though the total antibody levels may be similar, varying levels of antibodies which neutralize infectivity may contribute to differences between i.n. *C pne* versus *C. mur* immunized mice with respect to early resistance to i. vag. *C. mur* challenge.

We [14], [18], [21] and others [3] also have shown previously that the production of *Chlamydia*-specific IFN-γ also is crucial for the reduction of oviduct pathological sequelae following i.vag. *C. mur* challenge. In this study, we found that *C. pne* immunization induced a strong anti-*C. mur* IFN-γ response and a significant reduction in the incidence and severity of oviduct dilatation following i.vag. *C. muridarum* challenge. In comparison to *C. pne* immunization, intranasal immunization with live *C. muridarum* induced significantly greater anti-*C.mur* IFN-γ response and a further reduction, albeit not statistically significant, in oviduct pathology. These results demonstrate that a respiratory *C. pneumoniae* infection is capable of inducing a high level of protection, albeit arguably lesser than that induced by a prior *C. muridarum* infection, against oviduct pathological sequelae.

The results of this study provide a new perspective to our understanding of anti-chlamydial immunity. Specifically, (A) respiratory *C. pneumoniae* infection induces cross-species antigen-specific IFN-γ and antibody response against *C. muridarum* and *C. trachomatis*, and (B) respiratory *C. pneumoniae* infection induces significant reduction in the chlamydial shedding and oviduct pathology without affecting the incidence of infection upon i.vag. *C. muridarum* challenge. These should be considered in context of the observations that (C) intravaginal *C. muridarum* infection in mice is a reasonable model to study genital *C. trachomatis* infections in humans [1], [3] and (D) a significant number of children who contract *C. trachomatis* STI have been previously exposed to respiratory *C. pneumoniae* infection [12]. Collectively, it appears that prior exposure to *C. pneumoniae* infection may be an important factor in humans which may, at least partially, affect the duration of bacterial shedding and the development of pathological sequelae in females following genital *C. trachomatis* infection. The confirmation of such a possibility in studies involving humans also will have important implications to the approaches used for anti-chlamydial vaccine development.

In summary, we have used the mouse model to demonstrate a role for antecedent respiratory *C. pneumoniae* infection in altering the course of and sequelae to subsequent intravaginal *C. muridarum* infection. Furthermore, the demonstration of *C. pneumoniae* induced cross-species immune response against the highly prevalent *C. trachomatis* serovar D suggests the likelihood of a similar possibility in context of *C. trachomatis* STI in humans.
